# Molecular and morphological differentiation between *Aphis gossypii* Glover (Hemiptera, Aphididae) and related species, with particular reference to the North American Midwest

**DOI:** 10.3897/zookeys.459.7850

**Published:** 2014-12-01

**Authors:** Doris Lagos-Kutz, Colin Favret, Rosanna Giordano, David J. Voegtlin

**Affiliations:** 1 Illinois Natural History Survey, Prairie Research Institute, University of Illinois at Urbana-Champaign, 1816 S Oak Street, Champaign, IL 61820 USA; 2 University of Montreal, Department of Biological Sciences, Biodiversity Centre, 4101 rue Sherbrooke est, Montreal QC, H1X 2B2, Canada

**Keywords:** Aphid, host plant, morphology, phylogeny, sequence divergence, status novus

## Abstract

The cotton aphid, *Aphis gossypii*, is one of the most biologically diverse species of aphids; a polyphagous species in a family where most are host specialists. It is economically important and belongs to a group of closely related species that has challenged aphid taxonomy. The research presented here seeks to clarify the taxonomic relationships and status of species within the *Aphid gossypii* group in the North American Midwest. Sequences of the mitochondrial cytochrome oxidase 1 (COI), nuclear elongation factor 1-α (EF1-α), and nuclear sodium channel para-type (SCP) genes were used to differentiate between *Aphid gossypii* and related species. *Aphis monardae*, previously synonymised with *Aphid gossypii*, is re-established as a valid species. Phylogenetic analyses support the close relationship of members of the *Aphid gossypii* group native to North America (*Aphid forbesi*, *Aphid monardae*, *Aphid oestlundi*, *Aphid rubifolii*, and *Aphid rubicola*), Europe (*Aphid nasturtii*, *Aphid urticata* and *Aphid sedi*), and Asia (*Aphid agrimoniae*, *Aphid clerodendri*, *Aphid glycines*, *Aphid gossypii*, *Aphid hypericiphaga*, *Aphid ichigicola*, *Aphid ichigo*, *Aphid sanguisorbicola*, *Aphid sumire* and *Aphid taraxicicola*). The North American species most closely related to *Aphid gossypii* are *Aphid monardae* and *Aphid oestlundi*. The cosmopolitan *Aphid gossypii* and *Aphid sedi* identified in the USA are genetically very similar using COI and EF1-α sequences, but the SCP gene shows greater genetic distance between them. We present a discussion of the biological and morphological differentiation of these species.

## Introduction

Host plant association is often one of the main characters used to distinguish between closely related aphid species. However, host association can also be one of the main sources of misidentification of host-alternating aphids. These aphids migrate between taxonomically distant hosts, usually between woody and herbaceous plants. Taxonomic problems have been created when aphid morphs from primary (woody) host plants have been treated as separate species from those found living on secondary (herbaceous) or summer host plants. Host alternation provides an opportunity for aphids to acquire new hosts and may be a key to the rapid diversification of some groups of aphids (Eastop 1971, Dixon 1973, von Dohlen and Moran 2000), thereby leading to hard-to-distinguish species complexes. The evolution of *Aphis*, the largest aphid genus by a margin, is associated with the rapid diversification of herbaceous angiosperms (Heie 1996).

The *Aphis gossypii* group contains economically important and taxonomically problematic species, with *Aphid gossypii* Glover itself being the most biologically diverse and hence taxonomically challenging (Blackman and Eastop 2007). It has many different primary and secondary host plants and exhibits both holocyclic and anholocyclic life cycles (Kring 1955, Blackman and Eastop 2006, Margaritopoulus et al. 2006). Its taxonomic complexity is attested to by its 42 available synonyms, including the native North American species, *Aphis monardae* Oestlund (Favret 2014). Eastop and Hille Ris Lambers (1976) established this synonymy without comment. Lagos (2007) found that *Aphid monardae* is distinct morphologically from *Aphid gossypii* and treated it as a valid species. No type specimen of *Aphid monardae* could be found at the time, and no molecular or biological evidence was available to support this decision. The research presented here contains both molecular and biological evidence as well as an examination of material collected by Oestlund from *Monarda* spp.

In Europe, there are approximately 20 aphid species morphologically similar to *Aphid gossypii* (Stroyan 1984, Heie 1986). Several studies using mitochondrial, nuclear, and intron length polymorphism in the sodium channel para-type (SCP) genes have achieved some resolution discriminating *Aphid gossypii* and other *Aphis* species (Coeur d’acier et al. 2007, Foottit et al. 2008, Coccuzza et al. 2009, Carletto et al. 2009b, Kim et al. 2010a, Komazaki et al. 2010, Favret and Miller 2011). In North America, morphological studies show that species of the *Aphid gossypii* group can be misidentified easily (Voegtlin et al. 2004, Lagos 2007). The discrimination of species closely related to *Aphid gossypii* is of particular importance due to the recent introduction into North America of the soybean aphid, *Aphid glycines* Matsumura (Voegtlin et al. 2004). This species is obligately holocyclic and heteroecious, feeding on soybean, *Glycine max* (L.) Merr., as secondary host, and on *Rhamnus* spp. as primary host. *Aphis gossypii* has also been reported to colonize soybean in North America (Blackman and Eastop 2007), and while its colonization on soybeans is uncommon in the north central United States, some soybean-collected insect samples from Alabama, Georgia, Kansas, Louisiana, and Mississippi contained only *Aphid gossypii* or a mixture of both species (personal observation, Illinois Natural History Survey (INHS) insect collection records). These collections suggest that *Aphid gossypii* may be more common on soybeans in southern regions. There are no records of the exotic *Aphid nasturtii* Kaltenbach feeding on soybeans, and attempts to culture *Aphid nasturtii* on soybeans were not successful (David Ragsdale, personal communication); however, this species shares the primary host, *Rhamnus* spp., with both *Aphid glycines* and *Aphid gossypii*.

We here elucidate the phylogenetic relationship of species morphologically close to *Aphid gossypii* and the taxonomic status of *Aphid monardae* in the North American Midwest.

## Materials and methods

*Aphid collections*: Aphids were collected from their primary and/or secondary host plants from different sites in China, France, Italy, Japan, Spain and the USA, with the majority of the material originating from the Midwest of the USA. When possible, aphids were collected alive and reared on the host plant for the maturation of late instar nymphs. Adults were preserved in 95% ethanol and stored at -20°C until DNA extraction and microscope slide preparation. Collection data with INHS Insect Collection specimen voucher numbers are presented in Suppl. material 1.

*Morphology*: Archival microscope slides were prepared using the technique described by Pike et al. (1991). Individuals were selected from the same colonies as those selected for DNA extraction. Photographs of mounted specimens and measurements were taken using a Leica DM 2000 digital camera and SPOT Software 4.6 (Diagnostic Instruments, Inc). Analyses of variance of diagnostic characters, such as the distance from the base of the third antennal segment to the first secondary sensorium, the ratio of the lengths of the processus terminalis and the base of the sixth antennal segment, and the ratio of the lengths of the siphunculus and the cauda, were tested using JMP, Version 7 (SAS Institute Inc., Cary, NC, 1989–2007). Species identification of slide-mounted material was done by the first author, using published keys (Oestlund 1887, Gillette 1927, Hottes and Frison 1931, Palmer 1952, Kring 1955, Cook 1984, Voegtlin et al. 2004) and authoritatively identified specimens in the insect collections of the INHS and the University of Minnesota. Identifications of slide-mounted specimens were referenced to the aphid colony-mates used in the molecular analyses.

*DNA extraction, PCR amplification, and sequencing*: Two or three specimens per colony were sequenced individually. Individual specimens were crushed in a 1.5 ml microcentrifuge tube and DNA was extracted and purified using the QIAamp DNAmicrokit (QIAGEN Inc., Valencia, CA). The mitochondrial gene Cytochrome Oxidase I (COI) was amplified in two overlapping fragments: 5’ fragment with forward primer C1-J-1718 (Simon et al. 1994) and internal reverse primer C1-J-2411 (Lagos et al. 2012); 3’ fragment with internal forward primer C1-N-2509 (Lagos et al. 2012) and reverse primer TL2-N-3014 (Simon et al. 1994). The nuclear gene Elongation Factor-1-α (EF1-α) the following primers were used: EF3F (Lagos et al. 2012) and EF2 (Palumbi 1996). The length polymorphism of an intron in SCP was sequenced using the primers Aph13 and Aph15 (Carletto et al. 2009a). All primers were synthesized by Invitrogen™ Corporation (Carlsbad, CA). PCR used PuReTaq™ Ready-To-Go™ PCR 0.2 ml beads (GE Healthcare UK) mixed with 20 µl of PCR-grade water, 1 µl of F and R primers at 10 µM, and 3 µl of genomic DNA solution. The thermal cycler protocol used to amplify COI and EF1-α was: 95°C 2 min (95°C 30s; 53°C 30s; 72°C 120s) 40x. For SCP, it was: 95°C 3 min (94°C 60s, 55°C 45s, 72°C 60s) 40x. PCR products were run on a 1% agarose gel for 40 min at 90 v, and visualized with GelGreen nucleic acid stain (Biotium Inc, California, USA). Most PCR products were purified using QIAquick^®^ (QIAGEN Inc.) kit. PCR products that included the co-amplification of non-specific bands were gel purified using Zymoclean ™ gel DNA recovery kit (Zymo Research, USA). The concentration of PCR products was measured using a NanoDrop^®^ ND-1000 spectrophotometer (Thermo Fisher Scientific, Wilmington, DE). PCR products were sequenced in both directions using 3 µl of a mixture of BigDye^®^ Terminator v3.1, dGTP BigDye Terminator v3.0, and buffer in a ratio of 2:1:1 respectively, 1.6 µl of 2 µM primer primers, differing amounts of DNA, and 1 µl of dimethyl sulfoxide (DMSO) (SIGMA-ALDRICH^®^, St Louis, MO). Sequencing reactions were run using the following protocol: 96°C 2 min (95°C 20s; 50°C 5s; 60°C 240s) 25x. Sequencing reactions were cleaned using Performa^®^ DTR Ultra 96-Well Plates (EdgeBioSystems, Gaithersburg, MD) and run on ABI 3730 at the Keck Center (University of Illinois at Urbana-Champaign). Raw sequence data were examined and assembled using Sequencher 4.7 (Gene Codes Corporation, Ann Arbor, MI). Sequences were then aligned with Clustal X (version 2.0, 2007; Larkin et al. 2007). Three introns in EF1-α were identified and used in this study. Nucleotide sequences were deposited in GenBank (Suppl. material 1). Pairwise distances were obtained using PAUP 4.0b10 based on the Kimura two-parameter model (Swofford 2001).

The COI sequence of the *Aphid gossypii* neotype specimen (GU591547) and 25 EF1-α sequences of *Aphis* spp. (especially those of species closely related to *A. gossypii*) were retrieved from GenBank: EU019867, EU019869, EU019871, EU019872, EU019873, EU019874, EU019875, EU019876, EU019878, EU019879, EU358904, EU358907, EU358911, EU358915, EU358916, EU358917, EU358924, EU358926, EU358927, GU205375 and GU205376.

*Phylogenetic analysis*: Modeltest 3.7 (Posada and Crandall 1998) was used to select the best-fit nucleotide substitution model. Single sets of gene sequences were analyzed using MrBayes 3.1.2 (Huelsenbeck and Ronquist 2003) to execute Bayesian analyses. For single analysis, four chains were run. The number of generations was 5,000,000 with a burn-in of 250 trees and frequency sampling of 100 generations with rates equal to variable gamma as a model of substitution of nucleotides. *Rhopalosiphum maidis* (Fitch) (Aphidinae: Aphidini), and *Hyadaphis tataricae* Aizenberg and *Uroleucon helianthicola* (Olive) (Aphidinae: Macrosiphini) were selected as outgroups.

*Aphid* biology: Two growth chambers were used to examine various aspects of the biology of *Aphid monardae*, *Aphid gossypii*, and *Aphid sedi* Kaltenbach, in order to discern differences in their life cycle. Experimental plants were grown in a greenhouse in 12.7 cm diameter pots and isolated in 13.5 by 13.5 by 22.5 inches cages. Chamber A was set at 12°C and short photoperiod (8L:16D), conditions that will trigger the development of sexual morphs. Colonies of *Aphid monardae* on *Monarda fistulosa* L., *Aphid sedi* on *Hylotelephium telephium* (L.) H.Ohba, and *Aphid gossypii* on *Cucurbita pepo* L. and *Rhamnus cathartica* L. were exposed to these conditions for extended lengths of time. Samples of *Aphid monardae* and *Aphid sedi* were collected on a weekly basis from the host plants listed above and examined for the presence of sexual morphs. In the cages of *Aphid gossypii* weekly samples were taken from *Rhamnus cathartica*.

The B chamber was set at 24°C with constant illumination (24 hours) to keep colonies and test host plant specificity of the three species mentioned above. The following experiments were done in chamber B: a *Monarda fistulosa* plant infested with *Aphid monardae* was placed into a cage with an aphid-free *Cucurbita pepo* plant and left for a several weeks. Biweekly examination of the *Cucurbita pepo* plants was made to determine if *Aphid monardae* had colonized them. A *Cucurbita pepo* plant infested with *Aphid gossypii* was placed into a cage with aphid-free *Monarda fistulosa* and *Hylotelephium telephium* and left for several weeks. Biweekly examination of *Monarda fistulosa* and *Hylotelephium telephium* was made to see if *Aphid gossypii* had colonized them. A *Hylotelephium telephium* plant infested with *Aphid sedi* was placed into a cage with aphid-free *Cucurbita pepo* and left for several weeks. Biweekly examination of *Cucurbita pepo* was made to see if *Aphid sedi* had transferred to them. An entire tree of *Rhamnus cathartica* infested with *Aphid gossypii* was isolated in a 2 by 2 by 2-m walk-in cage in May of 2011 on the grounds of the South Farms of the University of Illinois (Suppl. material 1). The temperature ranged between 10 and 22 °C, http://www.isws.illinois.edu/atmos/statecli/cuweather/. Aphid-free *Cucurbita pepo*, *Hylotelephium telephium* and *Glycine max* were placed into the cage to document the potential infestation of these secondary hosts under natural environmental conditions.

## Results

### Phylogenetic analysis

A total of 160 COI sequences from 28 species, 133 EF1-α sequences from 36 species, and 13 SCP sequences from 6 species were used in this study. After alignment and excluding the primer sites, 1,290, 1,078 and 703 bp for COI, EF1-α (including gaps and introns) and SCP were used in the analysis, respectively. COI sequence divergence between species of the *Aphid gossypii* species group ranged from 0.08% (between *Aphid gossypii* and *Aphid sedi*) to 3.04% (between *Aphid gossypii* and *Aphid monardae*). The sequence divergence of *Aphid glycines* and *Aphid nasturtii* (sharing a winter host plant with *Aphid gossypii*), as compared with the species of the *gossypii* group, ranged from 5.25% (between *Aphid gossypii* and *Aphid glycines*) to 6.97% (between *Aphid nasturtii* and *Aphid sedi*) (Table 1). The sequence divergences of EF1-α and SCP are presented in Table 2. Generally, COI sequences were more conserved than EF1-α, which in turn were more conserved than SCP.

**Figure 1.**
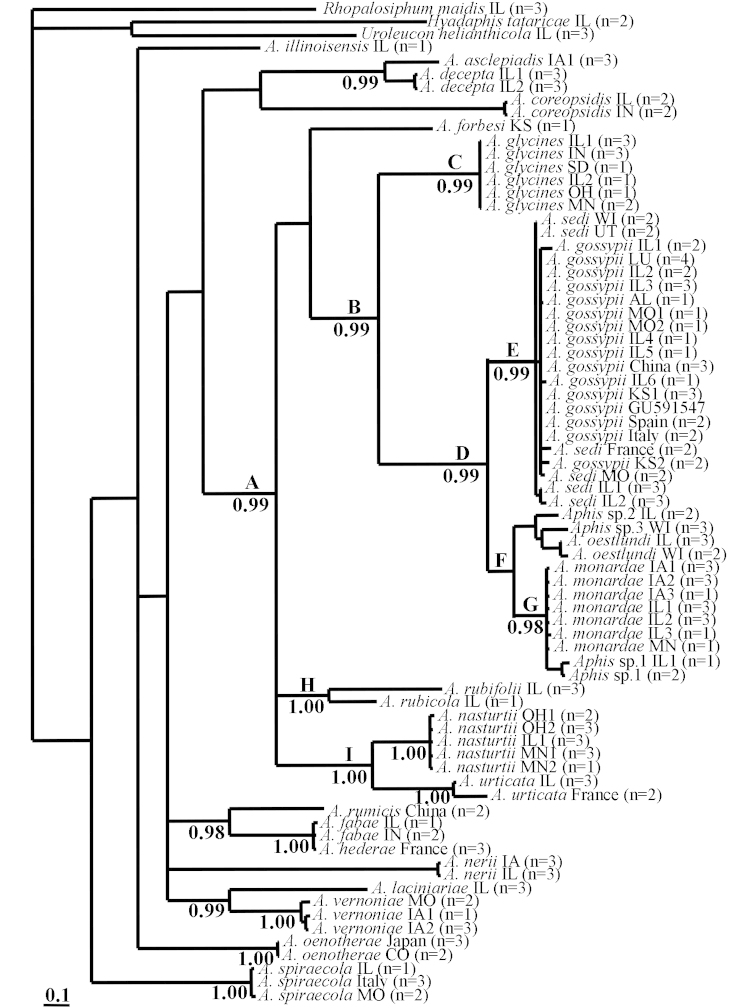
Cladogram inferred based on analysis of COI with MrBayes. Support values (Posterior Probabilities) are below branches. Values under 0.95 are not presented. Species names are followed by collection locality (USA: AL (Alabama), CO (Colorado), IA (Iowa), IL (Illinois), IN (Indiana), KS (Kansas), LA (Louisiana), MO (Missouri), MN (Minnesota), OH (Ohio), SD (South Dakota), WI (Wisconsin)), and number of haplotypes.

**Figure 2.**
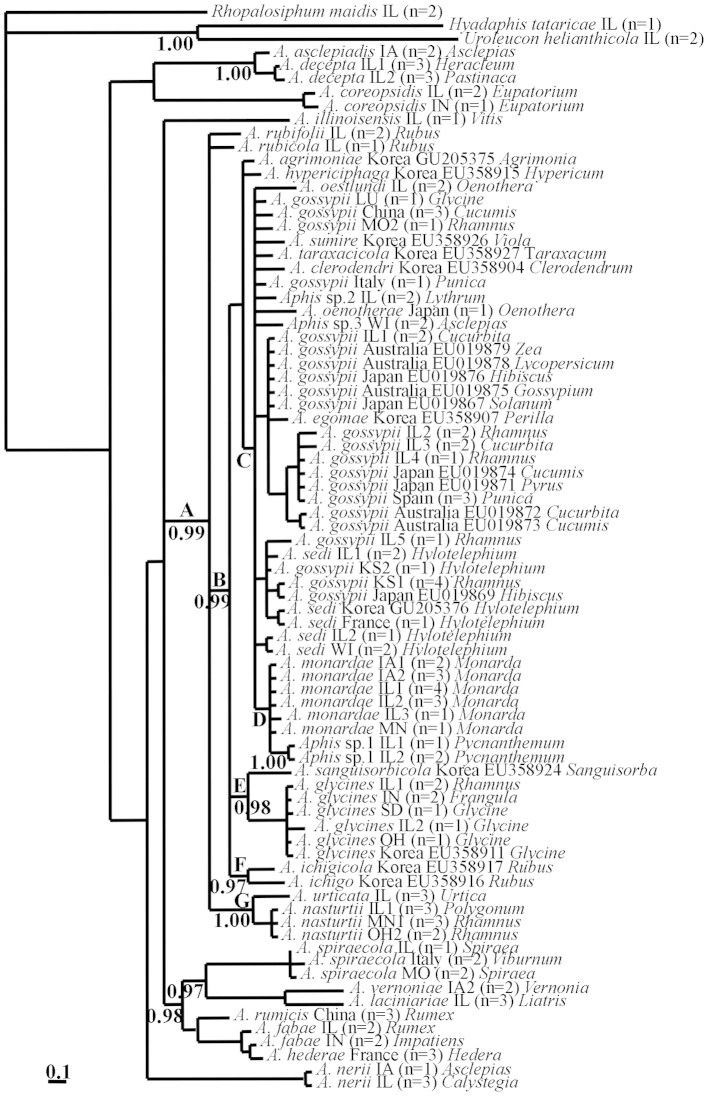
Cladogram inferred based on analysis of EF1-α with MrBayes. Support values (Posterior Probabilities) are below branches. Values under 0.95 are not presented. Species names are followed by collection locality, number of haplotypes and genus of host plant.

The cladograms using COI (Figure 1) and EF1-α (Figure 2) showed a high level of agreement. The COI analysis supported the monophyly of a group of species (Clade A) related to *Aphid gossypii*, including *Aphid glycines*, *Aphid nasturtii*, and a still more closely related group that are regarded as members of the *Aphid gossypii* complex (Clade D). Within Clade A are several supported groups: Clade H of *Aphid rubifolii* (Thomas) and *Aphid rubicola* Oestlund (PP:1.00); Clade I of *Aphid nasturtii* and *Aphid urticata* Gmelin (PP:1.00); Clade B of *Aphid glycines* with the *Aphid gossypii* complex (PP:0.99). The *Aphid gossypii* complex is itself well supported (Clade D, PP:0.99), and includese two groups: Clade E of *Aphid gossypii* and *Aphid sedi* (PP:0.99) and Clade F of *Aphid oestlundi* Gillette, *Aphid monardae*, and several possible new species.

The dendrogram inferred by MrBayes using EF1-α (Figure 2) is congruent with that of COI for some taxa mentioned above (clade A, PP:0.99), although lack of resolution prevented recovery of a monophyletic Midwest *Aphid gossypii* complex. The close relationship of *Aphid nasturtii* and *Aphid urticata* is robustly supported (Clade G, PP:1.00) as is clade B (PP:0.99). Clade A in the COI analysis is polyphyletic in the EF1-α analysis and includes Asian species *Aphid ichigicola* Shinji and *Aphid ichigo* Shinji (Clade F, PP:0.97); *Aphid glycines* and *Aphid sanguisorbicola* Takahashi (Clade E, PP:0.98). Clade C is poorly supported and presents polytomies of species closely related to *Aphid gossypii*: *Aphid sedi*, *Aphid oenotherae* Oestlund; the Asian taxa *Aphid egomae* Shinji, *Aphid sumire* Moritsu, *Aphid* taraxacicola (Börner), and *Aphid clerodendri* Matsumura; and the North American species *Aphid monardae* and *Aphid oestlundi*.

**Figure 3.**
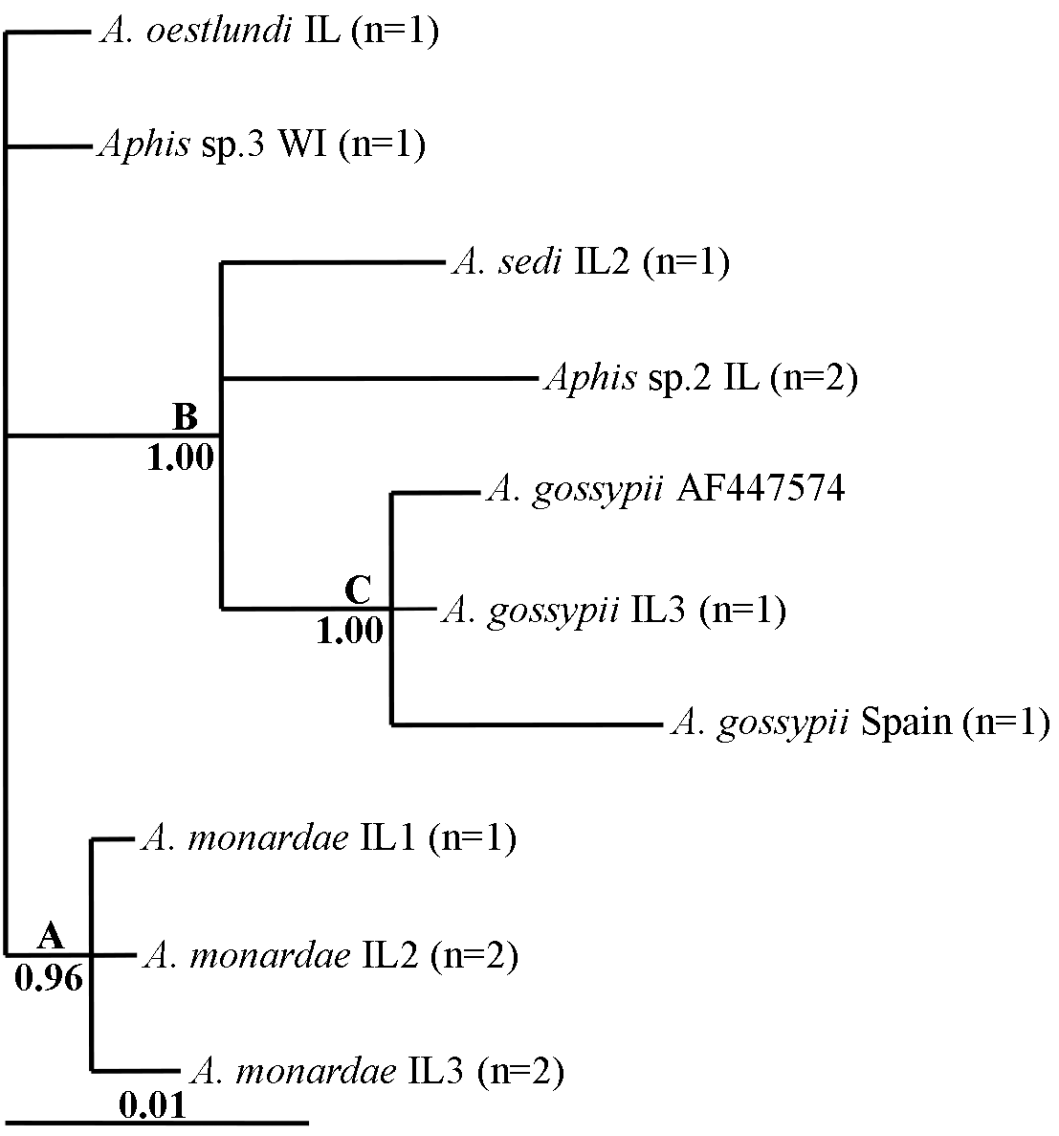
Inferred relationships using the SCP gene based on analysis with MrBayes. Support values (Posterior Probabilities) are below branches. Species names are followed by the collection locality (USA) and number of haplotypes.

The SCP gene was difficult to amplify and thus we only acquired sequences for six taxa. The Bayesian cladogram using SCP (Figure 3) shows two groups strongly supported: *Aphid monardae* (Clade A, PP: 0.96) and the group comprised of *Aphid sedi*, *Aphis* sp.2 and *Aphid gossypii* (Clade B, PP:1.00).

**Table 1.** Range of Kimura 2 Parameter pair-wise inter- and intraspecific sequence divergence (%) for COI sequences.

	*Aphid forbesi*	*Aphid glycines*	*Aphid gossypii*	*Aphid monardae*	*Aphid nasturtii*	*Aphid oestlundi*	*Aphid sedi*
*Aphid forbesi*	0.00						
*Aphid glycines*	5.49–5.73	0.00					
*Aphid gosyypii*	6.27- 6.35	5.25–5.92	0.00–0.54				
*Aphid monardae*	6.27	5.75–5.85	2.70–3.04	0.00–0.08			
*Aphid nasturtii*	5.73–5.77	7.03–7.15	6.66–6.89	6.50–6.73	0.00–0.08		
*Aphid oestlundi*	6.35	5.67–5.85	2.37–2.57	1.57–1.81	6.57–6.68	0–0.16	
*Aphid sedi*	6.2–6.35	5.51–5.76	0.08–0.70	2.62–3.02	6.54–6.97	2.37–2.77	0.00–0.54

**Table 2.** Range of Kimura 2 Parameter pair-wise inter- and intraspecific sequence divergence (%) for EF1-α and SCP sequences.

	***Aphid gossypii***		***Aphid monardae***		***Aphid oestlundi***		***Aphid sedi***	
	**EF1-α**	**SCP**	**EF1-α**	**SCP**	**EF1-α**	**SCP**	**EF1-α**	**SCP**
*Aphid gossypii*	0.40–0.87	0.14–0.84						
*Aphid monardae*	0.54–0.97	1.12–1.98	0.00–0.11	0.14–0.28				
*Aphid oestlundi*	0.76–1.20	1.12–1.83	0.87–0.98	0.42–0.64	0.00	0.00		
*Aphid sedi*	0.11–0.76	0.84–1.84	0.65–0.76	1.26	0.87	1.26	0.00–0.22	0.00

**Figure 4.**
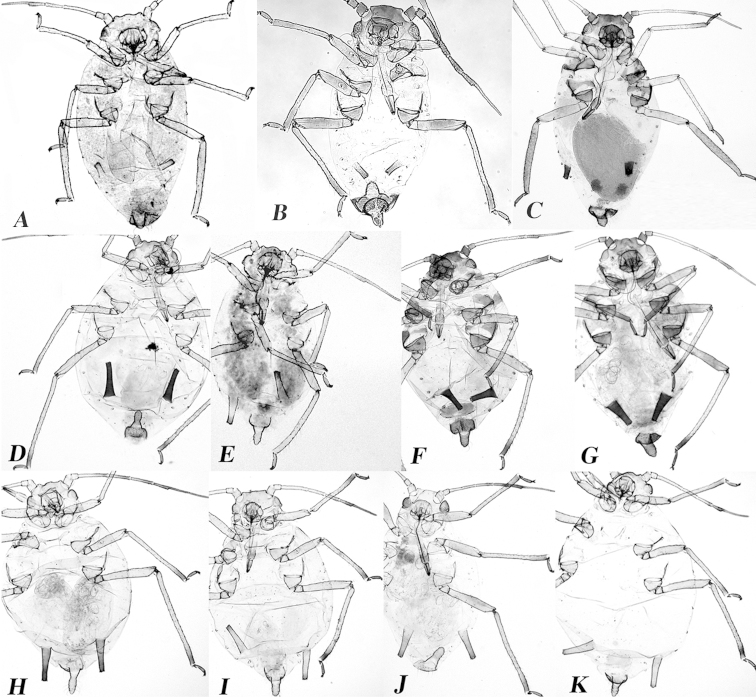
Habitus images of slide-mounted sexual morphs of **A** ovipara of *Aphid monardae* **B** male of *Aphid monardae* **C** ovipara of *Aphid sedi*, and apterous viviparae of **D** *Aphid gossypii* **E** *Aphid gossypii* **F** *Aphid gossypii* **G** *Aphid sedi* **H** *Aphid glycines* **I** *Aphid monardae* **J** *Aphid nasturtii* **K** *Aphid oestlundi*.

### Biological evidence

After four weeks under conditions of reduced temperature and photoperiod, colonies of *Aphid monardae* reared on *Monarda fistulosa* produced oviparae and apterous males (Figure 4A–B). Also, sexual morphs were collected in the field (Middlefork Savanna, Lake County) at the beginning of October (Suppl. material 1). *Aphis sedi* on *Hylotelephium telephium* produced oviparae (Figure 4C) but no males were found. Voucher slides of both species are deposited in the INHS insect collection with the following catalog numbers: *Aphid monardae*, 512858-512865; *Aphid sedi*, 511202-511208 and 511559-511573. In chamber A, no sexual morphs of *Aphid gossypii* were found after two months exposure to the low temperatures and reduced photoperiod and weekly collections on *Rhamnus cathartica*.

The outdoor experiments located at the South farms of the University of Illinois were evaluated after 25 days. Alate viviparae of *Aphid gossypii* were seen on *Hylotelephium telephium* and *Glycine max* but they did not produce offspring, however, alates that moved to *Cucurbita pepo* did produce apterous and alate viviparae. Voucher slides are deposited in the INHS insect collection numbers: 512851-512857. The colonies of *Aphid gossypii* reared on *Cucurbita pepo* were set in a growth chamber B where they grew rapidly. Potted *Monarda fistulosa* were placed in this chamber and were colonized by *Aphid gossypii*. Clean plants of *Cucurbita pepo* that were later exposed in the same chamber to a colony of *Aphid monardae* were not colonized. A colony of *Aphid sedi* begun with fundatrices from *Hylotelephium telephium* was exposed to *Cucurbita pepo* in growth chamber B for several weeks, but the aphids did not transfer to and establish on this plant.

**Figure 5.**
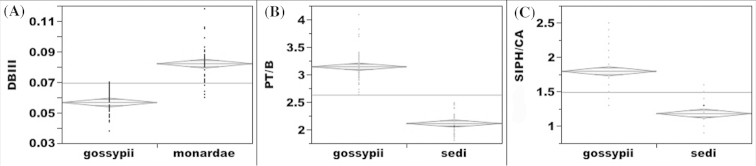
Analysis of variance of morphological characters useful to discriminate *Aphid gossypii*, *Aphid monardae*, and *Aphid sedi*. The gray line represents the median. The gray diamond represents the means and standard deviation. A 95% level indicates a significant difference. **A** distance from the base of antennal segment III to the first secondary sensorium (DBIII) between *Aphid gossypii* and *Aphid monardae* **B** ratio of length of processus terminalis (PT) to the base of last antennal segment **B** between *Aphid gossypii* and *Aphid sedi* **C** ratio of length of siphunculi (SIPH) to the length of cauda (CA) between *Aphid gossypii* and *Aphid sedi*.

### Comparison of *Aphis monardae* and *Aphis gossypii*

**Figure 6.**
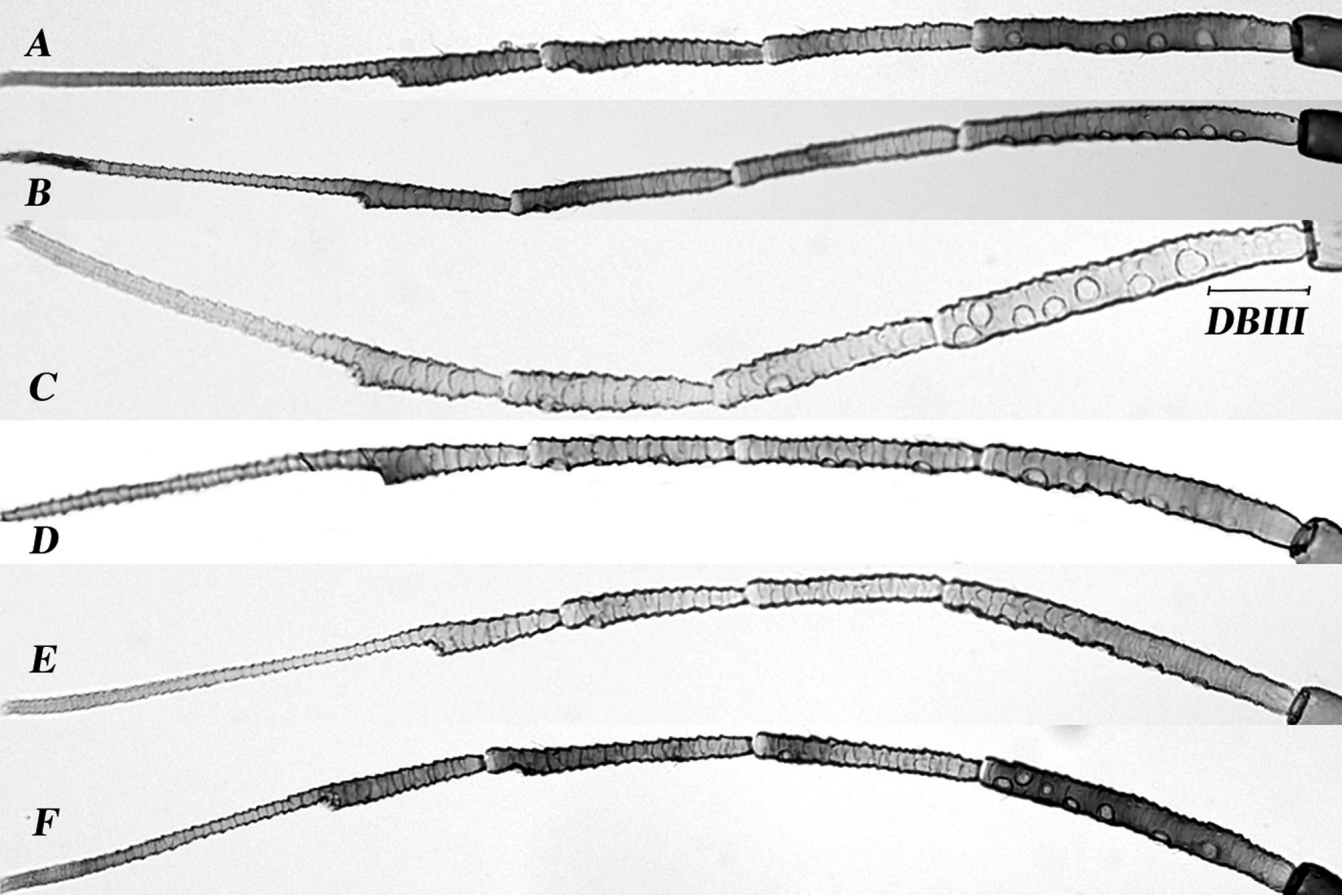
Antennal segments (II-VI) of alate viviparae **A** *Aphid glycines* **B** *Aphid gossypii* **C** *Aphid monardae*, showing distance from the base of antennal segment III to the first secondary sensorium, DBIII **D** *Aphid nasturtii* **E** *Aphid oestlundi* **F** *Aphid sedi*.

In both the COI and EF1-α analyses, *Aphid monardae* was readily distinguished from *Aphid gossypii* (Figure 1 Clade G, Figure 2 Clade D). *Aphis monardae* and *Aphid gossypii* are also differentiable morphologically: 1) the siphunculi of apterous morph are darker in *Aphid gossypii* than in *Aphid monardae*, and 2) and secondary sensoria on antennal segment IV are always absent in alate viviparae of *Aphid gossypii*, but present in *Aphid monardae* (Suppl. material 2). A third, novel morphological character, the distance from the base of antennal segment III to the first secondary sensorium (DBIII) in alate viviparae also separates these species consistently. In *Aphid gossypii*, the secondary sensoria are uniformly distributed along the segment (Figure 6B) but not in *Aphid monardae* (Figure 6C). The means of the distance from the base of antennal segment III to the basal margin of the first secondary sensorium of *Aphid gossypii* and *Aphid monardae* are 0.06 and 0.08 mm, respectively (Figure 5A, F ratio=152.3, df=1, P<0.0001). Evidence in support of the reproductive isolation of this species is the presence of oviparae (Figure 4A) and apterous males of *Aphid monardae* (Figure 4B) on *Monarda fistulosa* (INHS insect collection numbers: 511335-511344 and 512858-512865, respectively), as well as a COI sequence divergence of 2.7-3.04% between *Aphid gossypii* and *Aphid monardae* (Table 1).

### Redescription of *Aphis monardae* Oestlund, 1887

*Diagnosis*: Siphunculi of apterous morph pale, dark distally. When alive, light yellow to light green, body covered with white wax (Figure 8B). In alate viviparae: secondary sensoria on antennal segment IV present (Figure 6C). The distance from the base of antennal segment III to the first secondary sensorium (DBIII) 0.06-0.12 (0.08).

*Neotype*: Apterous viviparous female. USA: Minnesota; Douglas County; on *Monarda fistulosa* L.; 45.8160°N, 95.7472°W; 19.viii.2010; D. Lagos. Neotype apterous viviparous female (INHS Insect Collection 513070). Body1.4, URS 0.09, accessory setae 2, antennal segments: III 0.16, IV 0.08, V 0.09, B 0.08, Pt 0.18, LHIII 0.010, hind tibiae 0.50, HT2 0.08, width of tubercle on abdominal tergite I 0.020, width of tubercle on abdominal tergite VII 0.018, siphunculus 0.19, cauda 0.12, with 5 setae, abdominal tergite VIII with 2 setae, sub-genital fig with 3 setae on anterior part.

See Suppl. material 2 for morphological measurements of the four morphs of *Aphid monardae*. Additional images of *Aphid monardae* can be found in Lagos et al. (2014a).

*Apterous viviparae* (n= 40). *Color in life* (Figure 8B): Head, thorax and abdomen vary from light yellow to light green. *Color of cleared specimens* (Figure 4I): *Head*: dusky. All antennal segments pale, except the sixth throughout, which is dusky. Secondary sensoria absent. URS does not reach the hind coxae. *Thorax*: Coxae, trochanters and all femora dusky. All hind tibiae dusky and dark distally. *Abdomen*: Cauda slightly dusky, tongue-shaped. Siphunculi dusky and dark distally, imbricated with flange. Marginal sclerites pale. Marginal tubercles only present on abdominal segments I and VII. Dorsal abdomen without sclerites. Pre and post-siphuncular sclerites. Abdominal tergite VIII with 2 setae. Subgenital fig complete, slightly dusky with 2-7 setae on anterior part. Cuticle with reticulation.

**Figure 7.**
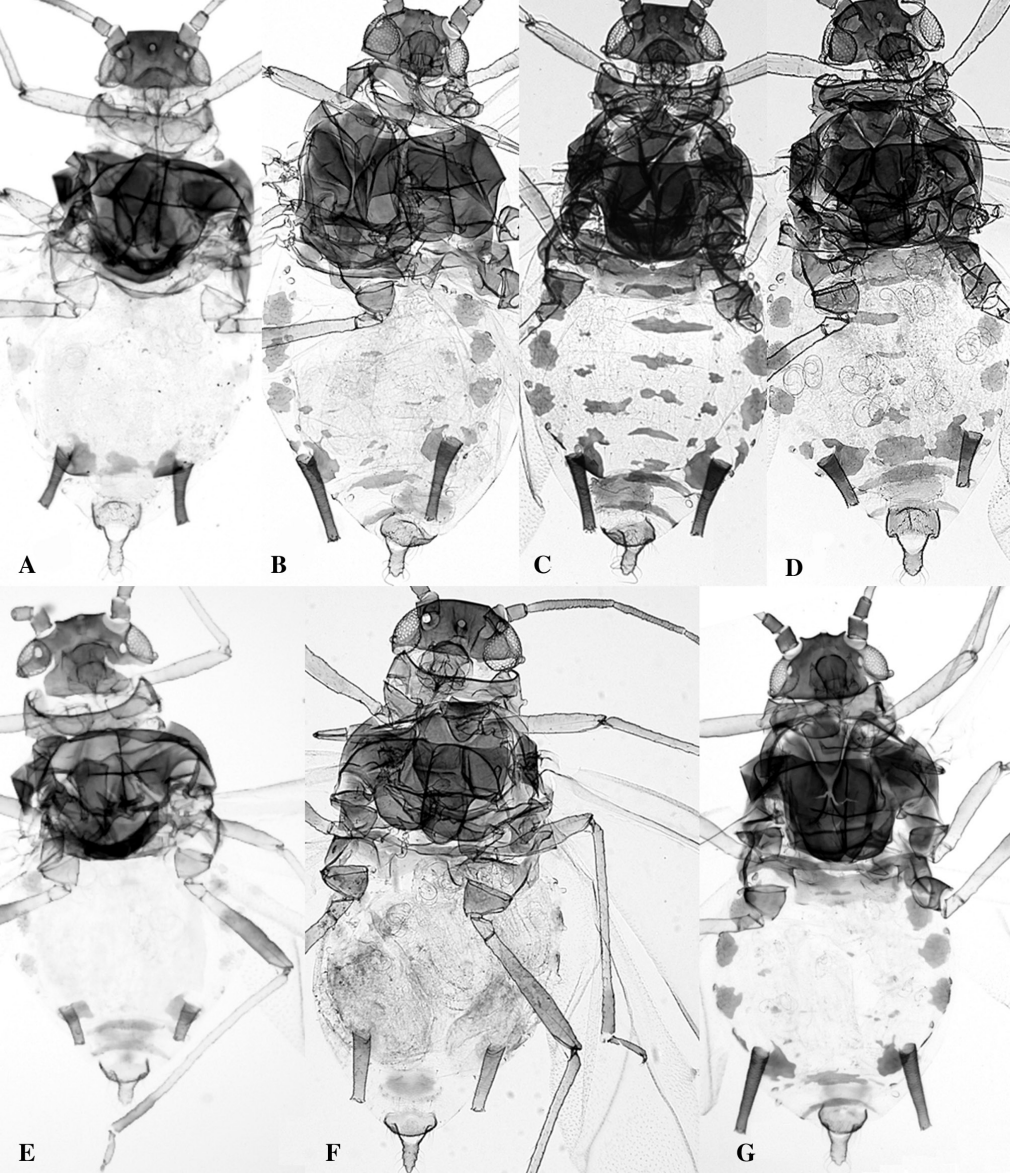
Body of alate viviparae. **A** *Aphid glycines* **B** *Aphid gossypii* **C** *Aphid gossypii* **D** *Aphid sedi* **E** *Aphid monardae* **F** *Aphid nasturtii* **G** *Aphid oestlundi*.

**Figure 8.**
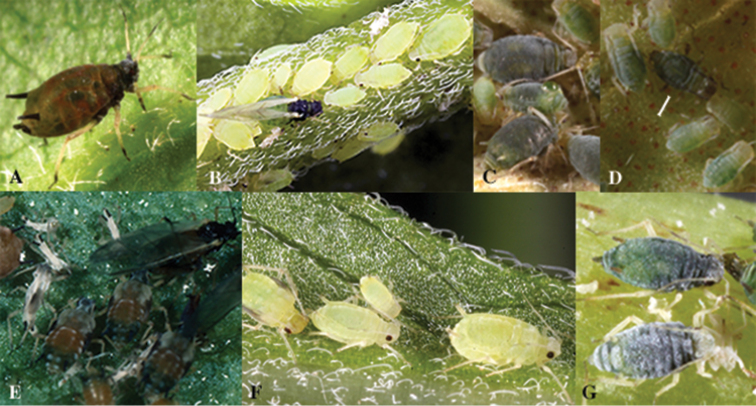
*Aphis* species of the *Aphid gossypii* complex. **A** Apterous vivipara of *Aphid gossypii* on *Rhamnus cathartica* **B** Nymphs, apterous and alate viviparae of *Aphid monardae* on *Monarda fistulosa* **C** Apterous ovipara of *Aphid monardae* **D** Nymphs and apterous male (brownish in the center of the image) of *Aphid monardae* **E** Nymphs and alate vivipara of *Aphid gossypii* on *Cucurbita pepo* **F** Nymphs and apterous vivipara of and *Aphid oestlundi* on *Oenothera biennis* **G** Apterous vivipara (top) and apterous ovipara (bottom) of *Aphid sedi* on *Hylotelephium telephium*.

*Alate viviparae* (n= 59). *Color in life* (Figure 8B): Head and thorax brown. Abdomen green. *Color of cleared specimens* (Figure 7E): *Head*: dark. Antennal segments: first and second dark, the rest dusky. Secondary sensoria present on and III and IV. Arrangement of secondary sensoria in a single row on the distal half (Figure 6C). *Thorax*: All femora dusky except in the base. Hind coxa dark. Hind trochanters paler than coxa. Hind tibiae dark distally. *Abdomen*: Cauda pale or slightly dusky. The cauda parallel-sided with constriction near the base. Siphunculi dark throughout,imbricated with flange. Pre-siphuncular sclerites absent. Post-siphuncular sclerites dusky. Marginal sclerites pale. Marginal tubercles only present on abdominal segments I and VII. Dorsal abdomen with small transverse sclerites on VI, VII and VIII. Abdominal tergite VIII with 2 setae. Subgenital fig complete, slightly dusky, with 2-7 setae on anterior part. Cuticle without reticulation.

*Oviparae* (n= 26). *Color in life* (Figure 8C): *Head*: varies from light brownish to dark green. Antennal segments: first, second and ¾ of third pale yellowish, the rest dusky. *Thorax*: Coxae and trochanters pale or dusky. Fore femora dusky throughout, mid-femora dusky except at base, hind femora dark except at base. Tibiae dusky distally and tarsi dusky. *Abdomen*: Cauda dark green. Siphunculi lighter than dark green abdomen. *Color on slide and morphological characters* (Figure 4A): *Head*: Dusky without frontal setae. Antennal tubercle undeveloped. Antennae five-six segmented, shorter than body. Antennal segments: first, second, third and four pale, the rest dusky. Rostrum reaches mesocoxae. *Thorax*: Coxae and trochanters dusky. All femora dusky throughout. Tibiae and tarsi dusky throughout. *Abdomen*: Cauda dusky, parallel-sided with blunt tip and bearing 6-8 setae. Siphunculi pale, smooth with flange. Pre and post-siphuncular sclerites absent. Marginal tubercles only present on abdominal segments I and VII. Dorsum of abdomen without sclerites. Abdominal tergite VIII with 4-8 setae. Subgenital fig dark, with 4-17 setae on anterior part. Cuticle without reticulation.

*Alate male* (n=17). *Color in life* (Figure 8D): *Head*: brownish. Antennae: blackish. *Thorax*: greenish. Legs light brown and tibiae distally dark as well as tarsi. *Abdomen*: Cauda dark green. Siphunculi lighter than dark green abdomen. *Color on slide and morphological characters* (Figure 4B): *Head*: dark. Antennae dark with secondary sensoria scattered on segments III, IV, and V. *Abdomen*: Cauda pale or dusky, parallel-sided with blunt tip and bearing 3-6 setae. Marginal tubercles present on abdominal segments I and VII. Dorsum of abdomen without large transverse sclerites. Male genitalia with 2 short claspers anteriorly and aedeagus centrally.

### Comparison of *Aphis sedi* and *Aphis gossypii*

The distinction of *Aphid sedi* from *Aphid gossypii* is supported by phenotypic characters of specimens in collections included in Tables S1 and S2. In addition, morphological characters such as the ratio of the lengths of the processus terminalis and the base of the sixth antennal segment (Suppl. material 2, Figure 5B: F ratio=498.1, df=1, P<0.001) and the ratio of the lengths of the siphunculus and the cauda (Suppl. material 2, Figure 5C: F ratio=168.5, df=1, P<0.001) of apterous viviparae can be useful to discriminate these species. Interestingly, only oviparae of *Aphid sedi* reared on *Hylotelephium telephium* were collected under laboratory conditions (Figures 4C and 8G). In contrast with the morphological differences, the interspecific genetic divergences using COI and EF1-α sequences of *Aphid gossypii* and *Aphid sedi* are less than 1% (Tables 1 and 2). SCP showed greater genetic divergence between these two species, namely 0.84–1.84% (Table 2).

### Comparison of *Aphis gossypii* with *Aphis forbesi*, *Aphis glycines* and *Aphis nasturtii*

These species are sometimes misidentified because they share some morphological characters on either apterous or alate morphs. Moreover, the pair-wise sequence divergences using COI sequences between *Aphid gossypii* and *Aphid forbesi* Weed, *Aphid glycines* and *Aphid nasturtii* are up to 5% (Table 1). Here we present some characters that can be useful for their discrimination. Apterous viviparae of *Aphid gossypii* can be differentiated from those of *Aphid forbesi* by the width of the marginal tubercles on abdominal segments I and VII (maximally 0.011 in *Aphid gossypii* and minimally 0.025 in *Aphid forbesi*; range for *Aphid gossypii* is given in Suppl. material 2), number of antennal segments and color pattern of siphunculi. Apterae of *Aphid gossypii* are differentiated from those of *Aphid glycines* by the shape of the cauda (Figures D-F, H) and the number of caudal setae, and from those of *Aphid nasturtii* by the absence of marginal tubercles on abdominal segments II and VI (Figure 4J). Alate viviparae of *Aphis gossypii* can be differentiated from those of *Aphid forbesi* by the number of secondary sensoria on III and the DBIII (Suppl. material 2), from those of *Aphid glycines* by the color of the hind coxae and marginal sclerites (Figure 7A) and from those of *Aphid nasturtii* by the number of secondary sensoria on antennal segments III, IV and V, absence of marginal tubercles on abdominal segments II and VI, and shape of cauda (Figure 7F). More figures and morphological characters have been uploaded in Lagos et al. 2014a.

### Dichotomous keys to apterous and alate viviparous females of the *Aphis gossypii* complex in the Midwest

Many dichotomous keys to subsets of *Aphis* have been written (Hottes and Frison 1931, Palmer 1952, Rojanavongse and Robinson 1977, Cook 1984, Stroyan 1984, Heie 1986, Brown 1989, García Prieto et al. 2005, Blackman and Eastop 2006) when morphological characters were not useful to discriminate between species, host plant associations have been used. Unfortunately, in the Midwest *Aphid gossypii* has been found on most of the host plants of other *Aphis* species included in this complex (*Aphid gossypii*, *Aphid monardae* stat. nov., *Aphid oestlundi* and *Aphid sedi*). The alternative key that we present below is based on specimens from collections made in the Midwest, and molecular data for specimens from these collections (Tables 1 and 2) supports our morphologically based identifications. Morphological data for these species is shown in Suppl. material 2. For some comparative morphometric data of European specimens of *Aphid gossypii* and *Aphid sedi* see Stroyan (1984), Heie (1986), Brown (1989) and García Prieto et al. (2005). The key is specific to Midwest collected specimens and may not be reliable in other geographic regions. It also demonstrates the difficulty of separating these closely related species using only morphological characters.

### Key to apterous viviparae

**Table:** 

1	Cauda pale, most often with constriction at midpoint, with 4–7 setae. Antennae five or six segmented. Siphunculi pale, distally dusky. Summer morphs. Polyphagous (Figure 4E)	***Aphid gossypii***
–	Cauda dusky or dark	**3**
3	Siphunculi dark all throughout	**4**
–	Siphunculi dusky or lighter at the base	**7**
4	Cauda constricted	**5**
–	Cauda not constricted	**7**
5	Cauda spoon-shaped, distinctly constricted, with 4–7 setae. Ratios PT/B 2.6–4.1, SIPH/CA 1.3–2.5. Polyphagous (Figure 4D)	***Aphid gossypii***
–	Cauda slightly constricted	**6**
6	Cauda slightly constricted at midpoint, with 4–5 setae. Ratios PT/B 2.0–2.7, SIPH/CA 1.5–2.2. On *Oenothera* spp. (Figure 4K)	***Aphid oestlundi***
–	Cauda elongate, parallel-sided, with acute tip and slight constriction at the base, and with 4–8 setae. Ratios PT/B 1.8–2.5, SIPH/CA 0.9–1.6. On *Hylotelephium* spp. (and elsewhere recorded from *Sedum* spp. and some other Crassulaceae) (Figure 4G)	***Aphid sedi***
7	Siphunculi lighter at the base, dusky distally. Cauda tongue-shaped, with 6–9 setae. Ratios PT/B 1.7–2.9, SIPH/CA 1.3–1.7. On *Monarda* spp. (Figure 4I)	***Aphid monardae***
–	Siphunculi dusky. Cauda tongue-shaped, with 4–7 setae. Ratios PT/B 2.6–4.1, SIPH/CA 1.3–2.5. Polyphagous (Figure 4F)	***Aphid gossypii***

### Key to alate viviparae

**Table:** 

1	Cauda tongue-shaped, with 3–9 setae, without sclerites on dorsum abdominal segments I, II, and III. Secondary sensoria on antennal segment III (4–9), IV (0–3). DBIII 0.07–0.12 (Figure 6C). Ratios PT/B 1.9–3, SIPH/CA 1.1–1.8. (Figure 7E)	***Aphid monardae***
–	Cauda constricted, sometimes with sclerites on dorsum of abdominal segments I, II, and III	**2**
2	Antenna VI PT/B 2.1–3.6. Secondary sensoria on antennal segment III (4–10) DBIII 0.04–0.07 (Figure 6B). Sometimes with transverse sclerites on dorsum of all abdominal segments (Figures 7B–C). Ratio SIPH/CA 1.1–2.3. Polyphagous	***Aphid gossypii***
–	Antenna VI PT/B 1.9–2.3. Secondary sensoria on antennal segment III (7–10) and IV (0–2) (Figure 6F). Sometimes with transverse sclerites on dorsum of all abdominal segments (Figure 7D). Ratio SIPH/CA 0.9–1.5. On *Hylotelephium* spp.	***Aphid sedi***
–	Antenna VI PT/B 2.2–2.9. Secondary sensoria on antennal segment III (2–8) (Figure 6E). Never with sclerites on dorsum of abdomen (Figure 7G). Ratio SIPH/CA 1.8–2.1. On *Oenothera* spp.	***Aphid oestlundi***

## Discussion

The analysis of different species included in this study largely corroborates the results obtained by Coeur d’acier et al. (2007), Kim and Lee (2008), Kim et al. (2010a), Kim et al. (2010b), Kim et al. (2011) and Lagos et al. (2014b). The *gossypii* complex in the North American Midwest contains the following native species, *Aphid oestlundi* and *Aphid monardae*, and the invasive species *Aphid gossypii* and *Aphid sedi*. Collection host records for *Aphid gossypii* show that it has been collected on *Oenothera* and *Monarda*, the host plants of the native *Aphis* species listed above (Blackman and Eastop 2006). Collection records for the native species suggest a very limited host range, in contrast with the highly polyphagous *Aphid gossypii*. Our results indicate that these species can be differentiated by morphological characters as well as host association. Data from this study confirms the finding of Lagos (2007) that *Aphid monardae* is a valid species and not a synonym of *Aphid gossypii* (Eastop and Hille Ris Lambers 1976). The novel character (distance from the base of antennal segment III to its first secondary sensorium, DBIII) is useful to differentiate alate viviparae of *Aphid monardae* and *Aphid gossypii* when they are collected together in traps. The sexual morphs collected on *Monarda* under laboratory and field conditions indicate that *Aphid monardae* has a monoecious holocyclic life cycle. A neotype of *Aphid monardae* has to be designated according to the Article 75.3 of the International Code of Zoological Nomenclature (International Commission on Zoological Nomenclature 1999). Concomitant with the redescription of the species, we here designate a neotype of *Aphid monardae* from the state of Minnesota on *Monarda fistulosa*. Slides deposited by O.W. Oestlund in the Insect Collection of the University of Minnesota show collection data no earlier than 1896. However, the first description of *Aphis monardae* was published in 1887 and the original description specified neither a type nor the type locality (Oestlund 1887). The comparison of apterae, alatae and oviparae of Oestlund’s collections match the morphological characters of those collected recently (Suppl. material 1). Some slides made by Oestlund were remounted in 1968 so it was possible to better see the characters. For a neotype we chose a more recently collected specimen taken in Minnesota as it more clearly shows color pattern and other characters used in the redescription.

The discrimination of *Aphid gossypii* and *Aphid sedi* is clear when the aphids are alive (Figure 8). The identification problem arises when we examine samples that have lost their color by being stored in ethanol. Molecular data also are helpful. The pair-wise sequences divergences between these species using SCP are higher than for COI and EF1-α sequences (Tables 1 and 2). This marker also successfully differentiated the cryptic species *Aphid gossypii* and *Aphid frangulae* (Carletto et al. 2009a). Results obtained in this study corroborate the biological and morphological findings of Kring (1955), who found that *Aphid sedi* is holocyclic monoecious on *Hylotelephium*. In this study, only apterous oviparae were collected under laboratory conditions conducive to the production of sexuales (Figure 4C). Kring’s morphological observations showed that the ratio of the processus terminalis to the base of the last antennal segment (PT/B), and the ratio of the length of the siphunculus to the length of the cauda (SIPH/CA), are both greater in *Aphid gossypii* than *Aphid sedi* for all morphs (Suppl. material 2). Although the above characters are useful to differentiate these species, their identification (especially the alate viviparae) is still problematic because of their similar morphology and because these ratios overlap (Figures 5B–C).

The inclusion of *Aphid glycines*, *Aphid gossypii* and *Aphid nasturtii* in strongly supported clades (Clade A, Figures 1 and 2) is consistent with the findings of Foottit et al. (2008) but in disagreement with those of Kim et al. (2010a). Interestingly, these three invasive species share a winter host plant, *Rhamnus* spp., but this is not the only known overwintering host for *Aphid gossypii*. This indiscriminate behavior, in addition to multiple species sharing winter hosts, raises the possibility of interspecies hybridization (Müller 1986, Rakauskas 2003). Hybridization may or may not be successful but should be detectable in studies of gene flow and phenotypic characterization of putative hybrids.

The species regarded here as members of the *Aphid gossypii* complex, *Aphid gossypii*, *Aphid sedi*, *Aphid oestlundi* and *Aphid monardae* (Clade D), exhibit interesting biological, morphological and molecular patterns. *Aphis gossypii* has been shown to colonize numerous secondary host plants including those of closely related taxa (Stroyan 1984, Heie 1986, Blackman and Eastop 2006). Moreover, it is one of the few *Aphis* species with multiple primary host plants (Blackman and Eastop 2006). By contrast, the native taxa related to it and found in the Midwest have or are presumed to have monoecious holocyclic life cycles (see Suppl. material 1 for host plant information). *Aphis oestlundi*, *Aphid monardae* and *Aphid sedi* have wingless males, a characteristic that would contribute to the genetic isolation of these species. These sibling species possess morphological characters useful for diagnostic purposes (Suppl. material 2) and the values that support interspecific sequences divergences (Table 2) are similar to those found by Foottit et al. (2008) and Favret and Miller (2011). The identification of species related to *Aphid gossypii* is made more difficult because they feed on host plants that can also serve as host to *Aphid gossypii*. Interestingly, however, their colors in life differ and can be useful for identification. For example, *Aphid gossypii* is dark green or light brownish and its siphunculi are dark throughout (Figures 8A, E), although this can vary in summer dwarf specimens. *Aphis gossypii* is mostly darker than *Aphid monardae*, which is light yellow or green (Figures 8B, C), and *Aphid oestlundi* is light yellow (Figure 8F). The color of *Aphid sedi* is dark green (Figure 8G), like *Aphid gossypii*, although it has more white wax on its body (García Prieto et al. 2005).

The COI sequence divergence values obtained in this study are similar to those obtained in other studies (Cocuzza et al. 2009, Cognato 2006, Coeur d’acier et al. 2007, Foottit et al. 2008, Favret and Miller 2011, Wang and Qiao 2009). Moreover, the low pair-wise sequence divergences found between some species such as *Aphid gossypii* and *Aphid sedi* (Table 1) are consistent with those obtained by other workers such as Piffaretti et al. (2012). While COI data have been found useful to discern the phylogenetic relationships of many taxa, the use of COI sequence divergences to set cut-off points that can differentiate *Aphis* species should be used with caution, since it may lead to the misidentification of new species, a conclusion drawn by other studies for several orders of insects (Blaxter 2004, Hebert et al. 2004, Nadler 2002, Will and Rubinoff 2004, Smith et al. 2008).

Our work suggests the possible existence of three undescribed Midwestern species (*Aphis* spp. 1, 2, and 3) within the *gossypii* complex. Further studies need to be done to validate their status. It is likely that additional new species will be found within this group as material is gathered from a larger geographical area and combined molecular, morphological and biological data are used to analyze the new taxa. The use of multiple primary hosts is unusual for any species, thus lineages within the *gossypii* complex that select and limit themselves to specific hosts may be driving the speciation process within this group (Peccoud et al. 2010, Kim et al. 2011).

## Supplementary materials

Supplementary material 1 Table S1. Collection information. Data type: species data Explanation note: Collection information for specimens included in this study. INHS voucher and GenBank accession numbers are for specimens originating from a specific collection. This dataset is made available under the Open Database License (http://opendatacommons.org/licenses/odbl/1.0/). The Open Database License (ODbL) is a license agreement intended to allow users to freely share, modify, and use this Dataset while maintaining this same freedom for others, provided that the original source and author(s) are credited.Doris M. Lagos, Colin Favret, Rosanna Giordano, David J. Voegtlin

Supplementary material 2 Table S2. Morphological characters useful to discriminate *Aphid gossypii*, *Aphid monardae*, *Aphid oestlundi* and *Aphid sedi*. Data type: measurement data Explanation note: Morphological characters useful to discriminate *Aphid gossypii*, *Aphid monardae*, *Aphid oestlundi* and *Aphid sedi*. For all measurements and counts the range is given and the mean is in parentheses. All measurements in mm. Abbreviations: B base of last antennal segment, CA cauda, DBIII: Distance from the base of antennal segment III to the first secondary sensorium, HT2 second hind tarsus, LHIII longest Hair on ant. segm. III, PT: Processus terminalis, SIPH siphunculi, URS ultimate rostral segment. Data of oviparae of *Aphid gossypii* from Kim et al. (2010a). This dataset is made available under the Open Database License (http://opendatacommons.org/licenses/odbl/1.0/). The Open Database License (ODbL) is a license agreement intended to allow users to freely share, modify, and use this Dataset while maintaining this same freedom for others, provided that the original source and author(s) are credited.Doris M. Lagos, Colin Favret, Rosanna Giordano, David J. Voegtlin

## References

[B1] Blackman RL Eastop VF (2006) Aphids on the World’s Herbaceous Plants and Shrubs. Vols 1 and 2. Wiley, Chichester and New York, 1439 pp [Revised and updated version is available at http://www.aphidsonworldsplants.info]

[B2] Blackman RL Eastop VF (2007) Taxonomic issues. In: van Emden HF Harrington R (Eds) Aphids as Crop Pests.CAB International, Oxfordshire, 1–29. doi: 10.1079/9780851998190.0001

[B3] Blaxter ML (2004) The promise of a DNA taxonomy. Philosophical Transactions of Royal Society of London B: Biological Sciences 359: 669–679. doi: 10.1098/rstb.2003.1447 10.1098/rstb.2003.1447 PMC1693355 15253352

[B4] Brown PA (1989) Keys to the alate *Aphis* (Homoptera) of northern Europe. British Museum (Natural History), London. Systematic Entomology 5: 1–29.

[B5] Carletto J Lombaert E Cchavigny P Brevault T Lapchin L Vanlerberghe-Masutti F (2009a) Ecological specialization of the aphid *Aphis gossypii* Glover on cultivated host plants. Molecular Ecology 18(10): 2192–2212. doi: 10.1111/j.1365-294X.2009.04190.x 10.1111/j.1365-294X.2009.04190.x 19635073

[B6] Carletto J Blin A Vanlerberghe-Masutti F (2009b) DNA-based discrimination between the sibling species *Aphis gossypii* Glover and *Aphis frangulae* Kaltenbach. Systematic Entomology 34(2): 307–314. doi: 10.1111/j.1365-3113.2008.00458.x

[B7] Cocuzza GE Cavalieri V Zappala L Barbagallo S (2009) Genetic relationships inside *Aphis frangulae/gossypii* group based on mitochondrial DNA sequences. Redia 92: 65–68.

[B8] Coeur d’acier A Jousselin AE Martin JF Rasplus JY (2007) Phylogeny of the genus *Aphis* Linnaeus, 1758 (Homoptera: Aphididae) inferred from mitochondrial DNA sequences. Molecular Phylogenetics and Evolution 3: 598–611. doi: 10.1016/j.ympev.2006.10.006 10.1016/j.ympev.2006.10.006 17113793

[B9] Cognato AI (2006) Standard Percent DNA Sequence Difference for Insects Does Not Predict Species Boundaries. Journal of Economic Entomology 99(4): 1037–1045. doi: 10.1603/0022-0493-99.4.1037 16937653 10.1603/0022-0493-99.4.1037

[B10] Cook EF (1984) *Aphis* (Homoptera: Aphididae) recorded from Compositae in North America, with a key to the species East of the Rocky Mountains and comments on synonymy and redescriptions of some little known forms. Annals of the Entomological Society of America 77: 442–449.

[B11] Dixon AFG (1973) Biology of Aphids. Edward Arnold, London, 58 pp.

[B12] Eastop VF (1971) Deductions from the present day host plants of aphids and related insects. The Royal Entomological Society of London 6: 157–178.

[B13] Eastop VF Hille Ris Lambers D (1976) Survey of the World’s Aphids. Dr. W. Junk b.v. Publishers, The Hague, 573 pp.

[B14] Favret C Miller GL (2011) The neotype of the cotton aphid (Hemiptera: Aphididae: *Aphis gossypii* Glover 1877. Proceedings of the Entomological Society of Washington 113(2): 119–126. doi: 10.4289/0013-8797.113.2.119

[B15] Favret C (2014) Aphid Species File. Version 5.0/5.0. http://Aphid.SpeciesFile.org [7 March 2014]

[B16] Foottit RG Maw HEL von Dohlen CD Hebert PDN (2008) Species identification of aphids (Insecta:Hemiptera:Aphididae) through DNA barcodes. Molecular Ecology Resources 8: 1189–1201. doi10.1111/j.1755-0998.2008.02297.x 10.1111/j.1755-0998.2008.02297.x 21586006

[B17] García Prieto F Tinaut Ranera A Pérez Hidalgo N Nieto Nafría JM (2005) Género *Aphis* Linnaeus, 1788. HemipteraAphididae III. Fauna Ibérica. Vol. 28. Museo Nacional de Ciencias Naturales, CSIC, Madrid, 30–173.

[B18] Gillette CP (1927) Notes on a few aphid species and the genus *Illinoia* Wilson. Annals of the Entomological Society of America 20(3): 344–348.

[B19] Hebert PDN Ratnasingham S de Waard JR (2004) Barcoding animal life cytochrome c oxidase subunit 1 divergences among closely related species. Proceedings of the Royal Society B: Biological Sciences 270(Supplement): 96–99. doi: 10.1098/rsbl.2003.0025 10.1098/rsbl.2003.0025 PMC1698023 12952648

[B20] Heie O (1986) The Aphidoidea (Hemiptera) of Fennoscandia and Denmark. III Family Aphididae: subfamily Pterocommatinae and tribe Aphidini of subfamily Aphidinae. Fauna Entomologica Scandinavica 3(17): 1–314.

[B21] Heie O (1996) . The evolutionary history of aphids and a hypothesis on the coevolution of aphids and plants. Bollettino di Zoologia Agraria e di Bachicoltura 28: 149–155.

[B22] Hottes FC Frison TH (1931) The plant lice, or Aphididae, of Illinois. Bulletin Illinois Natural History Survey 19: 121–447.

[B23] Huelsenbeck J Ronquist F (2003) MRBAYES 3.1.2: Bayesian phylogenetic inference under mixed models. Bioinformatics 19(2): 1572–1574. doi: 10.1093/bioinformatics/btg180 12912839 10.1093/bioinformatics/btg180

[B24] Kim H Lee S (2008) A molecular phylogeny of the tribe Aphidini (Insecta: Hemiptera: Aphididae) based on the mitochondrial tRNA/COII, 12S/16S and the nuclear EF1-α genes. Systematic Entomology 33(4): 711–721. doi: 10.1111/j.1365-3113.2008.00440.x

[B25] Kim H Hoelmer KA Lee W Kwon Y Lee S (2010a) Molecular and morphological identification of the soybean aphid and other *Aphis* species on the primary host *Rhamnus davurica* in Asia. Annals of Entomological Society of America 103(4): 532–543. doi: 10.1603/AN09166

[B26] Kim H Lee W Lee S (2010b) Morphometric relationship, phylogenetic correlation and character evolution in the species-rich genus *Aphis* (Hemiptera:Aphididae). PloS One, 5(7): 1–13. doi: 10.1371/journal.pone.0011608 10.1371/journal.pone.0011608 PMC2904707 20657654

[B27] Kim H Lee S Jang Y (2011) Macroevolutionary patterns in the Aphidini aphids (Hemiptera: Aphididae): Diversification, host association and biogeographic origins. PloS One 6(9): 1–17. doi: 10.1371/journal.pone.0024749 10.1371/journal.pone.0024749 PMC3174202 21935453

[B28] Komazaki S Shigehara T Toda S (2010) Diversity of Japanese *Aphis gossypii* and comparison with other *Aphis* species based on the mitochondrial cytochrome oxidase I sequence. Annals of the Entomological Society of America 103(6): 916–924. doi: 10.1603/AN10085

[B29] Kring JB (1955) Biological separation of *A. gossypii* Glover and *A. sedi* Kaltenbach. Annals of the Entomological Society of America 48: 442–444.

[B30] Lagos DM (2007) Species of the genus *Aphis* in the Midwestern States of United States of America. MS thesis, University of Illinois at Urbana-Champaign, Illinois.

[B31] Lagos, DM Puttler B Giordano R Voegtlin DJ (2012) A new species of *Aphis* (Hemiptera:Aphididae) in Missouri on St. John’s Wort, *Hypericum kalmianum*, and re-description of *Aphis hyperici* Monell. Zootaxa 3478: 81–92.

[B32] Lagos DM Dmitriev D Voegtlin DJ (2014a) An Interactive Key to the Aphids of *Midwestern* United States of America. http://imperialis.inhs.illinois.edu/lagos/ [8 April 2014]

[B33] Lagos DM Voegtlin DJ Giordano R (2014b) *Aphis* (Hemiptera:Aphididae) species groups found in the Midwestern United States and their contribution to the phylogenetic knowledge of the genus. Insect Science 21: 1–18. doi: 10.1111/1744-7917.12089 24302699 10.1111/1744-7917.12089

[B34] Larkin MA Blackshields G Brown NP Chenna R McGettigan PA McWilliam H Valentin F Wallace IM Wilm A Lopez R Thompson JD Gibson TJ Higgins DG (2007) Clustal W and Clustal X version 2.0. Bioinformatics 23(21): 2947–2948. doi: 10.1093/bioinformatics/btm404 17846036 10.1093/bioinformatics/btm404

[B35] Margaritopoulus JT Tzortzi M Zarpas KD Tsitsipis JA and Blackman RL (2006) Morphological discrimination of *Aphis gossypii* (Hemiptera: Aphididae) population feeding on Compositae. Bulletin of Entomological Research 96: 153–165. doi: 10.1079/BER2005410 16556336 10.1079/ber2005410

[B36] Müller FP (1986) The role of subspecies in aphids for affairs of applied entomology. Zeitschrift Fur Angewandte Entomologie 101: 295–303.

[B37] Nadler SA (2002) Species delimitation and nematode biodiversity phylogenies rule. Nematology 4: 615–625. doi: 10.1163/15685410260438908

[B38] Oestlund OW (1887) Synopsis of the Aphididae of Minnesota. Bulletin of the Geological and Natural History Survey of Minnesota 14: 17–56.

[B39] Palmer M (1952) Aphids of the Rocky Mountain Region, Vol. 5. Thomas Say Foundation, Denver, Colorado, 452 pp.

[B40] Palumbi SR (1996) Nuclei acids II: the polymerase chain reaction. In: Hillis DM Moritz C Zimmer E A (Eds) Molecular Systematics.2^nd^ Edn. Sinauer Associates, 205–247.

[B41] Peccoud J Simon J-C Dohlen C Coeur d’acier A Plantegenest M Vanlerberghe-Masutti F Jousselin E (2010) Evolutionary history of aphid-plant associations and their role in aphid diversification. Comptes Rendus Biologies 333(6-7): 474–487. doi: 10.1016/j.crvi.2010.03.004 20541159 10.1016/j.crvi.2010.03.004

[B42] Piffaretti J Vanlerberghe-Masutti F Tayeh A Clamens A-L Coeur d’acier AC Jousselin E (2012) Molecular phylogeny reveals the existence of two sibling species in the aphid pest *Brachycaudus helichrysi* (Hemiptera: Aphididae). Zoologica Scripta 41: 266–280. doi: 10.1111/j.1463-6409.2012.00531.x

[B43] Pike KS Boydston L Allison D (1991) Winged viviparous female aphid species associated with small grains in North America. Journal of the Kansas Entomological Society 63: 559–602.

[B44] Posada D Crandall KA (1998) Modeltest: testing the model of DNA substitution. Bioinformatics 14(9): 817–818. doi: 10.1093/bioinformatics/14.9.817 9918953 10.1093/bioinformatics/14.9.817

[B45] Rakauskas R (2003) Hybridization between *Aphis grossulariae* and *Aphis schneideri* (Sternorrhyncha: Aphididae): An experimental approach. European Journal of Entomology 96(4): 401–408.

[B46] Remaudière G Remaudière M (1997) Catalogue des Aphididae du monde-Catalogue of the world’s Aphididae [Homoptera, Aphidoidea]. INRA, Paris, 473 pp.

[B47] Rojanavongse V Robinson AG (1977) Species of *Aphis* (Homoptera: Aphididae) in Manitoba, with a key, and descriptions of new species. The Canadian Entomologist 109: 649–666. doi: 10.4039/Ent109649-5

[B48] Simon J-C Frati F Beckenbach A Crespi B Liu H Flook P (1994) Evolution, weighting, and phylogenetic utility of mitochondrial gene sequences and a compilation of conserved polymerase chain reaction primer. Annals of the Entomological Society of America 87(6): 651–701.

[B49] Smith MA Rodriguez JJ Whitfield JB Deans AR Janzen DH Hallwachs W Hebert PDN (2008) Extreme diversity of tropical parasitoid wasps exposed by iterative integration of natural history, DNA barcoding, morphology and collections. Proceedings of the National Academy of Sciences 105: 12359–12364. doi: 10.1073/pnas.0805319105 10.1073/pnas.0805319105 PMC2518452 18716001

[B50] Stroyan HLG (1984) Aphids-Pterocommatinae and Aphidinae (Aphidini). Handbooks for the Identification of British Insects 2(6): 1–232.

[B51] Swofford DL (2001) PAUP*: Phylogenetic Analysis Using parsimony (*and other methods), version 4. Sinauer Associates, Sunderland, Massachusetts.

[B52] Voegtlin DJ Halbert SE Qiao G (2004) A guide to separating *Aphis glycines* Matsumura and morphologically similar species that share its hosts. Annals of Entomological Society of America 97(2): 227–232. doi: 10.1603/0013-8746(2004)097[0227:AGTSAG]2.0.CO;2

[B53] von Dohlen CD Moran NA (2000) Molecular data supports a rapid radiation of aphids in the cretaceous and multiple origins of host alternation. Biological Journal of the Linnean Society 71: 689–717. doi: 10.1111/j.1095-8312.2000.tb01286.x

[B54] Wang J-F Qiao G-X (2009) DNA barcoding of genus *Toxoptera* Koch (Hemiptera: Aphididae): Identification and molecular phylogeny inferred from mitochondrial COI sequences. Insect Science 16: 475–484. doi: 10.1111/j.1744-7917.2009.01270.x

[B55] Will KW Rubinoff D (2004) Myth of the molecule DNA barcodes for species cannot replace morphology for identification and classification. Cladistics 20: 47–55. doi: 10.1111/j.1096-0031.2003.00008.x 10.1111/j.1096-0031.2003.00008.x 34892971

